# Effects of a smartphone-based stress management program on work performance, sick leave, and intention to leave among nurses during COVID-19 in Vietnam and Thailand: an analysis of secondary outcomes of a randomized controlled trial

**DOI:** 10.1093/joccuh/uiaf061

**Published:** 2025-10-23

**Authors:** Asuka Sakuraya, Thuy Thi Thu Tran, Narisara Sripo, Kazuhiro Watanabe, Kotaro Imamura, Plernpit Boonyamalik, Natsu Sasaki, Thanate Tienthong, Hiroki Asaoka, Mako Iida, Quynh Thuy Nguyen, Nga Thi Nguyen, Thai Son Vu, Thuy Thi Ngo, Tham Thi Luyen, Long Duc Nguyen, Nga Thi Viet Nguyen, Binh Thanh Nguyen, Yutaka Matsuyama, Yukie Takemura, Daisuke Nishi, Akizumi Tsutsumi, Huong Thanh Nguyen, Orawan Kaewboonchoo, Norito Kawakami

**Affiliations:** Department of Digital Mental Health, Graduate School of Medicine, The University of Tokyo, Tokyo, Japan; Faculty of Environmental and Occupational Health, Hanoi University of Public Health, Hanoi, Vietnam; Princess Agrarajakumari College of Nursing, Chulabhorn Royal Academy, Bangkok, Thailand; Department of Digital Mental Health, Graduate School of Medicine, The University of Tokyo, Tokyo, Japan; Department of Public Health, Kitasato University School of Medicine, Sagamihara, Japan; Department of Digital Mental Health, Graduate School of Medicine, The University of Tokyo, Tokyo, Japan; Faculty of Public Health, Mahidol University, Bangkok, Thailand; Department of Mental Health, Graduate School of Medicine, The University of Tokyo, Tokyo, Japan; Princess Agrarajakumari College of Nursing, Chulabhorn Royal Academy, Bangkok, Thailand; Department of Mental Health, Graduate School of Medicine, The University of Tokyo, Tokyo, Japan; Department of Mental Health, Graduate School of Medicine, The University of Tokyo, Tokyo, Japan; Faculty of Environmental and Occupational Health, Hanoi University of Public Health, Hanoi, Vietnam; Faculty of Social and Behavioural Sciences, Hanoi University of Public Health, Hanoi, Vietnam; Faculty of Environmental and Occupational Health, Hanoi University of Public Health, Hanoi, Vietnam; Nursing Department, Pho Noi Hospital, Hung Yen, Vietnam; Nursing Department, Pho Noi Hospital, Hung Yen, Vietnam; Board of Directors, Saint Paul Hospital, Hanoi, Vietnam; Nursing Department, Saint Paul Hospital, Hanoi, Vietnam; Nursing Department, Saint Paul Hospital, Hanoi, Vietnam; Department of Biostatistics, School of Public Health, Graduate School of Medicine, The University of Tokyo, Tokyo, Japan; Department of Nursing, University of Tokyo Hospital, Tokyo, Japan; Department of Mental Health, Graduate School of Medicine, The University of Tokyo, Tokyo, Japan; Department of Public Health, Kitasato University School of Medicine, Sagamihara, Japan; Faculty of Social and Behavioural Sciences, Hanoi University of Public Health, Hanoi, Vietnam; Faculty of Public Health, Mahidol University, Bangkok, Thailand; Department of Digital Mental Health, Graduate School of Medicine, The University of Tokyo, Tokyo, Japan

**Keywords:** COVID-19, digital mental health intervention, intention to leave, quality of nursing care, sick leave, on-the-job performance

## Abstract

**Objectives**: The purpose of this secondary analysis of data from a previous randomized controlled trial (RCT) was to investigate whether an internet-based cognitive behavioral therapy (iCBT) stress management program improved work performance, sick leave, and intention to leave among nurses in Vietnam and Thailand during the COVID-19 pandemic.

**Methods**: Full-time nurses were recruited from hospitals in Vietnam and Thailand. A 2-arm, parallel-group, RCT was conducted. The intervention groups were provided a 7-week self-guided iCBT program. As secondary outcomes, work performance (on-the-job performance and quality of nursing care), sick leave days, and intention to leave the profession and the organization were assessed at baseline and 3-month and 6-month follow-ups in each group.

**Results**: A total of 1203 participants were randomly allocated to the intervention and the control group. The program significantly improved on-the-job performance at 3-month follow-up (*P* = .0499), although the effect was nonsignificant at 6-month follow-up; Cohen’s *d* was 0.16 and 0.04, respectively. The program also significantly reduced sick leave at 6-month follow-up (coefficient = −0.21; 95% CI, −0.36 to −0.07; prevalence ratio 0.81), although the effect at 3 months was nonsignificant. However, the effects of the intervention on the quality of nursing care and the intention to leave the profession or the organization were not significant.

**Conclusions**: A smartphone-based iCBT stress management program improved on-the-job performance at 3-month follow-up and decreased sick leave days at 6-month follow-up among hospital nurses in Vietnam and Thailand during the COVID-19 pandemic.

## Teaser

Key points:

- Previous randomized controlled trials reported the effectiveness of internet-based cognitive behavioral therapy (iCBT) on the mental health of nurses or health care professionals in pandemics such as COVID-19.
- However, no previous study addressed the effectiveness of iCBT programs on improving work-related outcomes such as on-the-job performance, sick leave, or intention to leave of nurses under such circumstances.
- This study examined the effect of a 7-week self-guided iCBT program on these outcomes among nurses in Vietnam and Thailand during the COVID-19 pandemic, demonstrating significant effects on improving on-the-job performance and sick leave.
- Our research findings may help to improve these work-related outcomes among nurses during a pandemic.

## 1. Introduction

The pandemic of the novel coronavirus disease 2019 (COVID-19) had a major impact on the mental health of healthcare workers, in particular nurses.^1^^,^^2^ Among others, the work demands of patient care and a fear of COVID-19 infection led to a variety of mental health problems, such as depression, anxiety, post-traumatic stress symptoms, insomnia, and alcohol and substance use in this population.^1-3^ Higher presenteeism,^4^^,^^5^ productivity loss,^4^ increased sick leaves,^6^ and increased turnover intention^7^^,^^8^ were also observed among health care workers during and after the pandemic. The average monthly increase in all-cause sick leave during the pandemic was 2.2-2.8 percentage points among healthcare workers in primary or specialist care compared with the pre-pandemic period.^6^ A prevalence of intention to leave the job was reported as 38%, and that of the intention to leave the profession was 28% among nurses during the COVID-19 pandemic.^8^ It has been reported that poor mental health caused by the COVID-19 pandemic contributed to the deterioration of these work-related outcomes thereby affecting the quantity and quality of work of the nursing workforce,^9-12^ which might even hinder the capacity of health care delivery to the whole community.

A promising strategy to improve the mental health of nurses in the COVID-19 pandemic was an internet-based mental health intervention.^13^^,^^14^ Such an intervention is easy to deliver during a pandemic, when face-to-face communication is limited to prevent disease transmission.^15^ It also has the advantages of easy access, lower cost, and greater anonymity ^16^^,^^17^.

A limited number of randomized controlled trials (RCTs) tested the effectiveness of internet-based cognitive behavioral therapy (iCBT) on the mental health of nurses or health care professionals during the pandemic.^18-20^ Two studies reported favorable effects on depression and other mental health outcomes of a therapist-guided iCBT^19^ and a self-guided iCBT during the pandemic.^20^ Therefore, an iCBT program could be an effective intervention to improve mental health among nurses in a pandemic.

However, no previous study has addressed the effectiveness of iCBT programs on improving work performance, sick leave, or intention to leave of nurses during the COVID-19 pandemic. Before the pandemic, evidence on the effectiveness of iCBT programs on these work-related outcomes was inconsistent. Among health care staff, an RCT reported a nonsignificant effect of the iCBT on work functioning and work ability at the 6-month follow-up.^21^ For non–health care workers, another RCT showed that the iCBT for insomnia significantly improved presenteeism,^22^ although many other studies did not.^23-25^ Similarly, for health care staff, a previous RCT reported that an internet-based mindfulness-based self-help intervention had a nonsignificant effect on sick leave days.^26^ For non–health care workers, one RCT on an iCBT program reported a significant effect on sick leave,^24^ whereas other studies reported a nonsignificant^22^ or only a marginally significant effect.^23^ To our knowledge, no RCT has investigated the effect of iCBT on intention to leave among any types of workers, although previous studies reported the effect of a face-to-face, group-based CBT program on decreasing intention to leave.^27^^,^^28^ Given that poor mental health was a determinant of poor performance-related outcomes,^11^^,^^12^ sick leave,^10^ and intention to leave^9^ during the pandemic among health care workers, iCBT programs may have improved these outcomes among nurses during the COVID-19 pandemic by improving their mental health.^19^^,^^20^

The purpose of this secondary analysis of data from a previous RCT,^20^ called the “COping with COVID-19 helps Nurses be Active, Tough, and Smiling” (COCONATS) project, was to investigate whether a smartphone-based iCBT stress management program improved work performance, sick leave, and intention to leave among nurses in Vietnam and Thailand during the COVID-19 pandemic. We analyzed the data to examine the effects of this iCBT program on secondary outcomes, including work performance—assessed by on-the-job performance and self-reported quality of nursing care— the number of sick leave days, and intention to leave both the profession and the organization.^29^ Southeast Asia is one of the most highly populated regions with rapid aging of its population, and faced the challenge of a shortage of health care workers even before the COVID-19 pandemic.^30^^,^^31^ In this context it would be valuable to know whether an iCBT intervention improves these work-related outcomes among nurses in this region.

## 2. Methods

### 2.1. Trial design

This was a 2-armed RCT (allocation ratio: 1:1) examining the improvement of depression as a primary outcome and, as stated, work performance (on-the-job performance and quality of nursing care), sick leave, and intention to leave as secondary outcomes. The results of the iCBT program for 7 weeks were compared with results of a control group at 3-month and 6-month follow-ups among hospital nurses in Vietnam and Thailand. After the completion of an online survey at baseline, the nurses in each hospital were randomly allocated to the intervention or control groups. Randomization was administered by stratifying the participants by countries, hospitals, and depression severity at baseline. The Research Ethics Review Board of Graduate School of Medicine/Faculty of Medicine, the University of Tokyo (32021082NI-[1]) and the Ethical Review Board for Biomedical Research of Hanoi University of Public Health (353/2021/YTCC-HD3) and Mahidol University (MU MOU COA No. 2021-001) approved the study procedure. The study protocol was registered at the University Hospital Medical Information Network (UMIN) Clinical Trials Registry (UMIN-CTR, ID=UMIN000044145). The trial protocol is published elsewhere.^29^ This manuscript conforms to the Consolidated Standards of Reporting Trials (CONSORT) guideline.^32^

### 2.2. Participants

Participants were recruited from 6 hospitals across Vietnam and Thailand. In Vietnam, researchers at the Hanoi University of Public Health (HUPH) distributed the advertisement for the research project and documents explaining the purpose and procedure of the study, with the baseline questionnaire (February-March 2022) to the potential participants. Nurses who agreed to join the study signed an informed consent form and answered the baseline survey (March-April 2022). After the randomization, participants in the intervention group received the intervention for 10 weeks. For the first 7 weeks, they were notified weekly by email or message/chat app that a new module was available; after 7 weeks, they had access to the program for an additional 3 weeks. The follow-up surveys were administered at 3 months (July 2022) and 6 months (October 2022) after baseline. In Thailand, the main research team opened an online meeting to explain the aims and steps of the study to all potential participants (February-March 2022). In the meeting, nurses who agreed to participate in the project signed the online informed consent form and completed the baseline questionnaire (March-April 2022). Those unable to attend the meeting could request project documents or seek further information from the principal investigator or local collaborators during the application period. After the randomization, the intervention group received the intervention for 10 weeks. The follow-up surveys were administered at 3 months (July-August 2022) and 6 months (October-November 2022) after baseline.

The inclusion criteria at the baseline survey were: being employed full-time as a registered nurse at 1 of the 6 hospitals in Vietnam or Thailand, and able to access the internet via a mobile device such as a smartphone. The exclusion criteria were as follows: (1) sick or maternity leave at baseline, or planned to take maternity leave or to leave or change their job in the next 6 months; (2) were assistant nurses or helpers; (3) were nonregular or part-time employees; (4) experienced sick leave for 10 or more days for a physical or mental condition in the past 4 weeks; and (5) were receiving treatment for a mental health problem from any health care professional.

### 2.3. Interventions

A 7-week smartphone-based self-guided stress management program for nurses in the COVID-19 pandemic in Vietnam and Thailand, “the ABC Stress Management” – COVID-19 version, was developed. The program contains 7 modules. The modules were presented in a fixed order, with 1 module accessible per week, from Module 1 to Module 7. Each module requires 5-10 minutes to read. The modules focused on the following themes: a transactional model of stress and coping (Module 1), self-case formulation based on the cognitive behavioral model (Module 2), behavioral activation (Module 3), cognitive restructuring (Module 4), cognitive restructuring and relaxation (Module 5), problem-solving (Module 6), and stress and coping for better mental health in the COVID-19 pandemic (Module 7). We developed the modules 1-6 using the modules of a previous iCBT program.^33^ For specifically teaching awareness of stress and learning stress coping in the COVID-19 pandemic, the seventh module was newly developed. Full details of these programs are reported elsewhere.^20^^,^^29^

### 2.4. Control: treatment as usual

We did not provide any intervention program to the participants in the control group for the first 6 months. Participants in both the intervention and control groups were free to use any other available mental health service in their workplace as usual treatment. After the 6-month follow-up survey, intervention programs were provided for the control group.

### 2.5. Outcomes

All outcomes were measured at baseline and at 3-month and 6-month follow-ups. Participants had 10 weeks to complete the program. All of the following outcomes were translated into Vietnamese and Thai languages**.**

#### 2.5.1. On-the-job performance

On-the-job performance in the past 4 weeks was sought using 1 item adopted from the WHO Health and Work Performance Questionnaire (HPQ).^34^ For on-the-job performance, participants were asked to evaluate their work performance during the past 4 weeks. The item was measured on an 11-point scale ranging from 0 (worst possible work performance) to 10 (top work performance).

#### 2.5.2. Sick leave days

For sick leave days, participants were asked to report the number of days they missed because of problems with their physical or mental health, including only days missed for their own health, not someone else’s health, during the 4 weeks by using 1 item from WHO HPQ.^34^ In this study, 4 respondents answered above the upper limit of sick leave (more than 28), which were converted to 28.

#### 2.5.3. Quality of nursing care

The self-reported quality of nursing care was measured by a single question^35^: “How would you describe the quality of nursing care you delivered in the last month?” There were 4 response options: (1) Excellent, (2) Good, (3) Fair, and (4) Poor. Responses other than these 4 options were treated as missing values. The item was scored on a 4-point scale ranging from 1 (Poor) to 4 (Excellent). In this study, 2 respondents answered abnormal values (5), which were converted to missing.

#### 2.5.4. Intention to leave

Both intention to leave the profession and intention to leave the organization were assessed.^36^: Intention to leave the profession was gauged by asking, “How often during the course of the past 3 months have you thought about giving up nursing?” with 5 response options: (1) never, (2) sometimes in 3 months, (3) sometimes a month, (4) sometimes a week, (5) every day. Intention to leave the organization was assessed by asking, “How often during the course of the past 3 months have you thought about leaving the organization?” with the same 5 response options. Each item was scored on a 5-point scale ranging from 1 (never) to 5 (every day).

#### 2.5.5. Demographic variables

Demographic and occupational variables were assessed using a questionnaire, including gender (male, female, or others), age, and marital status (unmarried, having a spouse, or separated/divorced/widowed).

### 2.6. Sample size

The sample size in this study was calculated for the primary outcome (ie, depressive symptoms assessed by the Depression, Anxiety, and Stress Scale–21-item [DASS-21]), assuming that the effect size was set to 0.15. According to a previous RCT on an iCBT program, the effect size for work-related outcomes was from 0.14 to 0.16.^23^ Assuming that the effect size was set to 0.15, the original sample size would be adequate for analyzing work-related outcomes. An estimated post hoc power (1 − beta) was .74 if the effect size was small (0.15), assuming that alpha was less than .05 (2-tailed) and the number of respondents who were included in the analyses was 602 in the intervention and 601 in the control group, by using G*Power 3.1.9.7.^37^

### 2.7. Randomization

Eligible participants were randomly allocated to the intervention or control group. Stratified permuted-block randomization was used. Participants were stratified according to country (Vietnam or Thailand), 6 hospitals, and subgroups based on the cutoff point of mild depression by the DASS-21 scores at baseline (≥10 or <10) into 24 strata (2 × 6 × 2). An independent biostatistician generated a stratified permuted block random table. The assignment of participants using the random table was conducted by an independent research assistant at the Department of Mental Health, The University of Tokyo, blinded to the researchers. The stratified permuted-block random table was password-protected and blinded to the researchers. It was not possible to blind the results of the allocation to the participants and researchers because this trial offered a psychological intervention.

### 2.8. Statistical analysis

On-the-job performance, intention to leave, and quality of nursing care were almost normally distributed. A mixed modeling for repeated measures was used for the analysis. The indicator of the intervention’s effectiveness was determined by examining the interaction between the group (1 = intervention, 0 = control) and time. Variables used in the stratified randomization (eg, countries, hospitals, and subgroups based on the cutoff point of mild depression by the DASS-21) were entered as covariates. Intention-to-treat analysis was adopted. Using the MIXED procedure, missing values for outcomes at follow-up surveys were imputed by applying the restricted maximum likelihood estimation. Thus, all the participants who completed the baseline survey were included in this analysis. The effect size was estimated in 2 ways. First, we estimated a regression coefficient for a group (the intervention groups vs the control group) × time (baseline and 2 follow-ups) interaction using the MIXED procedure, which was converted to an effect size by dividing by a pooled SD, calculated as the SD of within-group differences between time points in the total sample. Second, we calculated Cohen’s *d* only among participants who completed the questionnaire survey at baseline and each follow-up, dividing the difference in within-group changes (between time points) between the intervention and control groups by the same pooled SD described above.

For sick leave, the distribution was skewed toward 0 value, and did not follow a normal distribution. Thus, we used a generalized mixed model based on the assumption of Poisson distribution to test the effects of the intervention compared with the control for 3- and 6-month follow-ups. The indicators of the intervention’s effectiveness and covariates used in the analyses were the same as those used in the mixed modeling analysis. In addition, we calculated the effect sizes (prevalence ratio) and the 95% CIs. Because the GENLINMIXED procedure could not impute missing values using maximum likelihood estimation, we conducted multiple imputation to impute some missing data for sick leave. For imputing those missing data, IBM SPSS Missing Values was used by making 50 pseudo-complete datasets, using Markov Chain Monte Carlo (MCMC) and regression model, with a restriction of an integer from 0 to 28. To inform the imputation, the model included the related variables (eg, covariates of age, gender, marital status, countries, hospitals where participants work, and subgroups based on the cutoff point of mild depression by the DASS-21, and other relevant outcomes). Accordingly, the generalized mixed model for sick leave included all the participants in the analysis, including those with deficits at each time point. All statistical analyses were conducted using SPSS Statistics version 29.0 (IBM Corp, USA).

## 3. Results

### 3.1. Characteristics of participants

The participant flowchart and baseline characteristics are shown in Figure 1 and Table 1 of Watanabe et al.^20^ Among the recruited nurses, 1321 (23.8%) participated in the baseline survey. After 118 were excluded, 1203 met the eligibility criteria. Finally, the 1203 participants were randomly allocated, with 602 in the intervention group and 601 in the control group. At the 3-month follow-up, the response rates were 87.5% and 85.7% in the intervention and control groups, respectively. The rates were 86.0% and 86.4% at the 6-month follow-up in the intervention and control groups, respectively. Watanabe et al^20^ report details of the participants’ baseline characteristics. Briefly, more than 85% of the participants (*n* = 1060) were female, 60.9% (*n* = 733) had a spouse, and the mean age of the intervention and control groups ranged from 36.42 to 36.95 years.

**Table 1:** Means and SDs of outcome variables at the baseline, and at the 3- and 6-month follow-ups.^a^

	**Control**			**Intervention**						**95% CI**	
	***n***	**Mean**	**SD**	***n***	**Mean**	**SD**		***n***	**Effect size** ^**b**^	**Lower**	**Higher**
**On-the-job performance**											
**Baseline**	514	8.13	1.17	505	8.11	1.21					
**3-month follow-up**	417	8.04	1.21	417	8.16	1.20		737	0.16	0.01	0.30
**6-month follow-up**	474	8.02	1.39	465	8.01	1.32		795	0.04	−0.10	0.18
**Sick leave days** ^**c**^											
**Baseline**	559	2.13	3.92	567	2.14	3.68					
**3-month follow-up**	436	0.81	2.45	445	0.82	2.49		832	0.003^e^	−0.13	0.14
**6-month follow-up**	506	0.73	2.75	507	0.53	1.87		947	−0.05^e^	−0.18	0.08
**Quality of nursing care** ^**d**^											
**Baseline**	548	2.90	0.54	554	2.90	0.62					
**3-month follow-up**	426	2.99	0.64	423	3.01	0.70		788	−0.001	−0.14	0.14
**6-month follow-up**	486	2.93	0.59	478	2.90	0.61		872	−0.03	−0.16	0.10
**Intention to leave the profession**											
**Baseline**	597	3.19	1.55	602	3.13	1.57					
**3-month follow-up**	515	3.56	1.36	524	3.50	1.45		1035	0.08	−0.04	0.20
**6-month follow-up**	518	3.28	1.52	517	3.12	1.55		1031	−0.01	−0.14	0.11
**Intention to leave the organization**											
**Baseline**	595	3.21	1.57	600	3.15	1.58					
**3-month follow-up**	516	3.63	1.37	524	3.59	1.46		1032	0.07	−0.06	0.19
**6-month follow-up**	519	3.36	1.53	517	3.19	1.55		1029	−0.04	−0.17	0.08

a Higher scores indicate greater sick leave days, higher on-the-job performance, higher intention to leave the profession or organization, and better quality of nursing care.

b Cohen’s *d* values between baseline and each follow-up were based on the scores of the respondents who completed both surveys. ^c^Values of more than 28 were set to 28.

d Abnormal values (5) were treated as missing.

e Cohen’s *d* values for sick leave were reported for comparability, despite the nonnormal distribution.

### 3.2. Effect of the intervention programs on work-related outcomes

Table 1 shows each outcome variable’s mean and SD at baseline and at 3-month and 6-month follow-up in the intervention and control groups. The intervention showed a small but significant effect size in improving on-the-job performance at 3-month follow-up compared with the control group (*d* = 0.16; 95% CI, 0.01-0.30).

Table 2 shows the estimated effect of the intervention on improving on-the-job performance, quality of nursing care, and the intention to leave from mixed model analyses. The program significantly improved on-the-job performance at 3-month follow-up (*t* = 1.96; *P* = .0499), although the effect was nonsignificant at 6-month follow-up (*t* = 0.45; *P* = .66). For quality of nursing care and the intention to leave, there were nonsignificant intervention effects at all time points, and effect sizes were also small.

**Table 2:** Effects of the intervention program on on-the-job performance, intention to leave, and quality of nursing care from mixed model analyses.

			**95% CI**				
	***n*** ^**b**^	**Coefficient** ^**c**^	**Lower**	**Higher**	***t***	***P***	**Estimated effect size** ^**d**^
**On-the-job performance**							
**3-month follow-up**	1172	0.17	0.0001	0.34	1.96	.0499	0.13
**6-month follow-up**		0.04	−0.14	0.22	0.45	.66	0.03
**Pooled** ^**a**^		0.01	−0.02	0.04	0.50	.62	
**Quality of nursing care**							
**3-month follow-up**	1197	0.01	−0.09	0.11	0.19	.85	0.01
**6-month follow-up**		−0.04	−0.12	0.05	−0.78	.44	−0.05
**Pooled**		−0.01	−0.02	0.01	−0.76	.45	0.13
**Intention to leave the profession**							
**3-month follow-up**	1203	0.07	−0.08	0.21	0.89	.38	0.05
**6-month follow-up**		−0.06	−0.20	0.08	−0.79	.43	−0.05
**Pooled**		−0.01	−0.03	0.02	−0.73	.47	
**Intention to leave the organization**							
**3-month follow-up**	1203	0.06	−0.09	0.21	0.81	.42	0.05
**6-month follow-up**		−0.08	−0.22	0.06	−1.10	.27	−0.07
**Pooled**		−0.01	−0.04	0.01	−1.05	.30	

a Mixed models for repeated measures conditional growth model analyses were conducted.

b Because missing values for outcomes at follow-up surveys were imputed using the MIXED procedure, all the participants who completed the baseline survey were used in this analysis.

c The models were adjusted for the variables used in the stratified randomization: country (Vietnam and Thailand), hospital (6 hospitals), and the severity of depression (≥10 or <10 on the DASS-21 score).

d Effect sizes were calculated by dividing the estimated effect by the pooled SD.

Table 3 shows the effects of the intervention program on sick leave from generalized mixed model analyses. The program showed a significant pooled effect for decreasing sick leave (coefficient = −0.03; 95% CI, −0.06 to −0.01; prevalence ratio 0.97; 95% CI, 0.95 to 0.99). Regarding the variance model, the effect at 6-month follow-up was also significant (coefficient = −0.21; 95% CI, −0.36 to −0.07; prevalence ratio 0.81; 95% CI, 0.70 to 0.93), although the effect at 3-month follow-up was nonsignificant (coefficient = −0.01; 95% CI, −0.14 to 0.12; prevalence ratio 0.99; 95% CI, 0.87 to 1.13).

**Table 3:** Effects of the intervention program on sick leave from generalized mixed model analyses.^a^

				**95% CI**			**95% CI**	
	**N**	**Coefficient** ^**b**^	**SE**	**Lower**	**Higher**	**Prevalence ratio**	**Lower**	**Higher**
**Sick leave days**								
**3-month follow-up**	1203	−0.01	0.07	−0.14	0.12	0.99	0.87	1.13
**6-month follow-up**		−0.21	0.07	−0.36	−0.07	0.81	0.70	0.93
**Pooled**		−0.03	0.01	−0.06	−0.01	0.97	0.95	0.99

a For sick leave, a generalized mixed model based on the assumption of Poisson distribution was used.

b The models were adjusted for the variables used in the stratified randomization: country (Vietnam and Thailand), hospital (6 hospitals), and the severity of depression (≥10 or <10 on the DASS-21 score).

## 4. Discussion

The present secondary analysis of the data from an RCT showed that the smartphone-based iCBT program significantly improved on-the-job performance at 3-month follow-up among nurses in Vietnam and Thailand during the COVID-19 pandemic. However, the effect size was small (Cohen’s *d* = 0.16), indicating limited clinical significance of the program. The iCBT program also showed a statistically significant intervention effect on decreasing sick leave days at 6-month follow-up. The program did not show a significant intervention effect on the quality of nursing care or intention to leave the profession or the organization.

Most previous RCTs,^21^^,^^23-25^ except for one for insomnia,^22^ failed to show a significant effect of iCBT on performance-related outcomes, including on-the-job performance, in the pre-pandemic period. Our study indicated a significant intervention effect on on-the-job performance at 3-month follow-up. A possible reason for this positive finding is attributable to a strong association between poor mental health and decreased on-the-job performance during the pandemic.^11^^,^^12^  ^20^ Since the association may have been stronger in the pandemic period, improvement of depression with the iCBT may result in greater improvement in on-the-job performance in this study. A further analysis should be done to clarify the underlying mechanism, including mediation analyses. It should also be noted that the effect size was small (Cohen’s *d* = 0.16). This was smaller than the effect size reported in a previous systematic review of iCBT programs on work effectiveness (Hedges’ *g* = 0.26).^14^

Another pandemic-specific reason may be that because deterioration of on-the-job performance was possibly greater in the pandemic^4^^,^^5^ participants may have been more likely to respond to the intervention. A high program completion rate in this study may also have contributed to a positive finding: completion rates for each module of the intervention program, based on the log recorded on the server, ranged from 87.5% (527/602) to 74.3% (447/602); approximately 68.1% (410/602) of participants completed all 7 modules.^20^ A larger sample size in this study may also have given greater statistical power to detect the effect compared with previous studies.^21^^,^^24^^,^^25^ The effect of the intervention diminished at the 6-month follow-up, as did the effect on depression.^20^ The intensity of the intervention may not have been enough to maintain the effect for a longer period.

The number of sick leave days decreased in the intervention group more significantly than in the control group at the 6-month follow-up in this study. The finding is consistent with previous iCBT studies of non–health care workers pre-pandemic.^23^^,^^24^ Our study replicated these previous findings among hospital nurses in the COVID-19 pandemic. We did not identify the causes of sick leave in this study, and could not differentiate the observed effect into one on sick leave due to COVID-19 infection and that due to non–COVID-19 causes. In the United Kingdom^10^ it was reported that sick leave due to mental disorders increased among health care workers in the pandemic, aside from sick leave directly caused by COVID-19. We suppose that the intervention may prevent sick leave due to poor mental health. However, better preventive behaviors entailed by improved mental health due to the intervention may decrease the risk of COVID-19 infection and the associated sick leave. It would be interesting to investigate cause-specific reduction of sick leave days followed by an iCBT intervention in future studies.

The present study failed to show a significant intervention effect on the self-reported quality of nursing care and intention to leave. Despite improvement of on-the-job performance in the intervention group, the participants did not feel that their quality of nursing care was improved, under the high work demands of the COVID-19 pandemic^38^^,^^39^; it took great efforts to maintain nursing care quality at a level similar to that of the pre-pandemic period. It was also possible that the quality of nursing care was more strongly influenced by structural (eg, nurse staffing) and organizational factors, rather than by the mental health status of individual nurses.^35^ Similarly, the intention to leave the job or profession may also be more affected by organizational factors rather than the mental health status of individual nurses.^40^ A combination of an organizational-level intervention aimed at improving nurse staffing and workload, and/or a supportive working environment, with an individual-level intervention such as the present one, may achieve better outcomes.^41^

### 4.1. Limitations

This study has several limitations. First, the present study was conducted in large provincial hospitals in Thailand and Vietnam, low- and middle-income countries in Southeast Asia, during the COVID-19 pandemic. The findings may be different if it was done in smaller medical facilities. The findings also may not be generalized to other settings, such as lower- or higher-income countries, countries in other regions or with different cultures, and to the nonpandemic context. Second, dropouts may have caused an attrition bias. Although the follow-up rates were relatively high at each follow-up in both groups, if unmotivated participants in the intervention group dropped out, the effectiveness of the program would be overestimated. Third, this study could not blind the participants to the group. The bias in the outcome measurement may be caused if the participants in the intervention group were more motivated to report their on-the-job performance or intention to leave at more or less than the true level, respectively, at the follow-ups. Fourth, there may have been an underestimation of the intervention effect if the intervention group had shared information about the program content with the control group. Fifth, all outcomes in the current study were assessed by self-report and may have been influenced by participants’ perceptions and situational factors in the workplace. Self-reported measurements are susceptible to the Hawthorne effect, as well as biases due to recall and social desirability. A future study should consider adopting objectively measured outcomes such as nursing performance evaluated by patients or hospital records of nurses’ sick leave. Sixth, we did not assess how much participants understood the content of the iCBT program. In future work, it would be desirable to evaluate in more detail whether participants acquired the intended knowledge and attitudes from the program, since this could affect the outcomes. Seventh, although completion rates for each module of the iCBT were high (87.5% to 74.3%), 31.9% (192/602) of participants in the intervention group did not complete all 7 modules of the iCBT program.^20^ This may have attenuated the observed intervention effects.

## 5. Conclusions

The present secondary data analysis of an RCT indicates that a smartphone-based iCBT stress management program significantly improved on-the-job performance at 3-month follow-up and sick leave days at 6-month follow-up among hospital nurses in Vietnam and Thailand during the COVID-19 pandemic. However, the effect size for on-the-job performance was small, and the result may not be clinically meaningful. A self-guided smartphone-based stress management program may also be effective for improving on-the-job performance and preventing sick leave days among nurses in a pandemic, possibly through improving their mental health.

## Data Availability

The data related to the present study are available from the corresponding author, N.K., upon reasonable request. After considering ethical issues, the authors will approve a proposal.
