# Correction to: Effects of a smartphone-based stress management program on work performance, sick leave, and intention to leave among nurses during COVID-19 in Vietnam and Thailand: an analysis of secondary outcomes of a randomized controlled trial

**DOI:** 10.1093/joccuh/uiag059

**Published:** 2026-10-11

This is a correction to: Asuka Sakuraya, Thuy Thi Thu Tran, Narisara Sripo, Kazuhiro Watanabe, Kotaro Imamura, Plernpit Boonyamalik, Natsu Sasaki, Thanate Tienthong, Hiroki Asaoka, Mako Iida, Quynh Thuy Nguyen, Nga Thi Nguyen, Thai Son Vu, Thuy Thi Ngo, Tham Thi Luyen, Long Duc Nguyen, Nga Thi Viet Nguyen, Binh Thanh Nguyen, Yutaka Matsuyama, Yukie Takemura, Daisuke Nishi, Akizumi Tsutsumi, Huong Thanh Nguyen, Orawan Kaewboonchoo, Norito Kawakami, Effects of a smartphone-based stress management program on work performance, sick leave, and intention to leave among nurses during COVID-19 in Vietnam and Thailand: an analysis of secondary outcomes of a randomized controlled trial, *Journal of Occupational Health*, Volume 67, Issue 1, January-December 2025, uiaf061, https://doi.org/10.1093/joccuh/uiaf061

In the originally published version of this manuscript, the ‘Introduction’ contained a sentence that read: “The excess monthly rate of all-cause sick leave was 2.2%-2.8% among health care workers compared with the pre-pandemic rate”.

The sentence has been corrected to read: “The average monthly increase in all-cause sick leave during the pandemic was 2.2–2.8 percentage points among healthcare workers in primary or specialist care compared with the pre-pandemic period”.

Additionally, in the ‘Results’ section, there was an error which saw the number of female participants originally reported as 1061, with 60% having a spouse.

The relevant sentence has been corrected to read:

“Briefly, more than 85% of the participants (*n* = 1060) were female, 60.9% (*n* = 733) had a spouse, and the mean age of the intervention and control groups ranged from 36.42 to 36.95 years”.

